# Health and Healthcare Utilization among Asylum-Seekers from Berlin’s LGBTIQ Shelter: Preliminary Results of a Survey

**DOI:** 10.3390/ijerph17124514

**Published:** 2020-06-23

**Authors:** Nora Gottlieb, Conny Püschmann, Fabian Stenzinger, Julia Koelber, Laurette Rasch, Martha Koppelow, Razan Al Munjid

**Affiliations:** 1 Department of Health Care Management, Technical University Berlin, 10623 Berlin, Germany; 2 Berlin School of Public Health, Charité-Universitätsmedizin Berlin, 10117 Berlin, Germany; conny.pueschmann@charite.de (C.P.); fabian.stenzinger@charite.de (F.S.); julia.koelber@charite.de (J.K.); laurette.rasch@charite.de (L.R.); martha.koppelow@charite.de (M.K.); razan.almunjid@charite.de (R.A.M.)

**Keywords:** asylum-seekers, refugees, LGBTIQ, healthcare utilization, Germany, mental health, chronic illness, intersectionality, cross-sectional survey

## Abstract

Background: LGBTIQ asylum-seekers face multiple health risks. Yet, little is known about their healthcare needs. In 2016, Berlin opened the only major shelter for LGBTIQ asylum-seekers in Germany. This preliminary study describes health and healthcare utilization by asylum-seekers living in Berlin’s LGBTIQ shelter. To identify particular healthcare needs, we compared our results to asylum-seekers from other shelters. Methods: We surveyed residents of the LGBTIQ shelter and 21 randomly selected shelters in Berlin, using a validated questionnaire in nine languages (n = 309 respondents, including 32 respondents from the LGBTIQ shelter). Bivariate tests and generalized linear mixed models were applied to examine differences in health and healthcare utilization between the two groups. Results: Residents of the LGBTIQ shelter show high rates of chronic and mental illness. They use ambulatory and mental health services more frequently than asylum-seekers from other shelters, including a significantly higher chance of obtaining psychotherapy/psychiatric care in case of need. Emergency room utilization is also higher in the LGBTIQ group. Conclusions: Asylum-seekers from the LGBTIQ shelter face high chronic and mental health burdens. Tailored services in the LGBTIQ shelter help obtain adequate healthcare; they should be scaled up to maximize their potential. Yet, unmet needs remain and warrant further research.

## 1. Introduction: The Health of LGBTIQ Asylum-Seekers—Little Do We Know

Germany is legally committed to ensure the basic needs of asylum-seekers and refugees, and to protect vulnerable individuals among this group [1]. People who define themselves as lesbian, gay, bisexual, transsexual, intersexual, or queer (in the following: LGBTIQ) have become widely accepted as vulnerable in the public health discourse of the global North. Special attention to particular social and health needs has been recommended [2,3]. 

### 1.1. Asylum-Seekers’ Health and Healthcare in Germany

However, comprehensive information on healthcare needs among the asylum-seeking population in Germany is lacking. Existing studies are limited by low case numbers and regional particularities [4]. They indicate low subjective health [5,6], and prevalence rates of 40% for chronic diseases [6,7] and up to 45% for depression and anxiety [5,7]. Unspecific symptoms that are often related to psychological distress, such as back pain and headaches, are highly prevalent and have been linked to somatization [8]. Asylum-seekers were shown to frequently seek healthcare from general practitioners, yet some studies point to underutilization of specialist health services [6,7,8,9]. High hospitalization rates, including high rates of ambulatory care-sensitive hospitalizations, have been reported [6,7]. There is evidence for within-group disparities in health and access to healthcare [10].

These disparities have also been related to system-immanent healthcare barriers. Asylum-seekers’ health entitlements are restricted during their first 18 months in Germany (or until refugee status is granted) [11]. Implementation of the legal provisions is at the local authorities’ discretion. De facto healthcare provided for asylum-seekers thus varies across Germany [11,12] and prescribed treatments may not always be granted. For example, Baron & Flory [13] have documented that 49% of applications for psychotherapy coverage by asylum-seekers are rejected (as compared to 6% for statutorily insured persons). Low availability of health services can particularly affect asylum-seekers. As regards mental health, for instance, they can, in theory, seek the services of specialized “Psychosocial Centers for Refugees and Victims of Torture” (PSZs) or of registered psychotherapists. Both options are limited due to low availability. The PSZs report waiting times of up to two years and a 40% rejection rate [13]. Communication barriers and negative attitudes of administrative and healthcare staff have been described as additional deterrents to healthcare-seeking [14,15]. 

### 1.2. LGBTIQ Asylum-Seekers’ Health in Germany

The health of LGBTIQ asylum-seekers and refugees (for reasons of readability, in the following, the term “asylum-seekers” will be used to denote both asylum-seekers and refugees) is practically un-researched in the German context [16]. Information on sexual orientation and identity is neither collected within the framework of routine monitoring activities, nor as part of tailored health surveys. This reluctance may stem from good intentions to avoid exposure and stigmatization of LGBTIQ individuals. However, “if compulsory heterosexuality ‘others’ queer populations, then counting queer populations may undermine this ‘otherness’ by demonstrating the legitimate needs of the LGBTIQ* population for basic facilities” [17]. 

One study on health and healthcare utilization among lesbian women in Germany included migrants. While the authors found no differences by migration status, they refrained from drawing generalized conclusions due to high heterogeneity among the study population [18]. Media reports suggest that LGBTIQ asylum-seekers frequently suffer from abuse in refugee shelters (see, e.g., [19]). Further evidence from the German context is lacking [20,21]. 

### 1.3. Insights From the General LGBTIQ Health Literature

For the general LGBTIQ population, a growing body of literature demonstrates worse health, particularly in relation to mental health. Homosexual persons face a 2- to 3-fold risk of psychological or emotional problems and substance abuse, and a 1.5-fold risk of anxiety, depression, and suicidality compared to matched heterosexual comparisons. Risks are higher for trans- and intersex persons [22,23,24]. Elevated risks among some LGBTIQ subgroups are also reported for certain forms of cancer [22], cardiovascular disease [25], and HIV [26,27,28]. Upon seeking healthcare, LGBTIQ persons are more likely to encounter deterrents and barriers, such as stigma and discriminatory attitudes among staff [22,29]. 

The LGBTIQ health literature shows that certain subgroups among the LGBTIQ population face particular adversities in health and healthcare access, depending on sociodemographic, gender-related, and other groupings [22,30]. Elevated health risks have been related to minority stress, accumulated experiences of victimization and discrimination throughout the life course, heteronormativity and heterosexism, and stigma. Yet, evidence gaps remain on how different factors interact and create excess risks for some LGBTIQ subgroups, including asylum-seekers [22,30,31,32].

The few existing qualitative studies on LGBTIQ asylum-seekers’ health are focused on mental health. They explore the impact of recurrent trauma related to gender-based discrimination and violence in home countries [33,34], and how continued re-traumatization extends into post-migration contexts [35,36]. It has further been pointed out that precarious legal status and LGBTIQ identity intersect and compound difficulties in obtaining adequate healthcare [37]. 

### 1.4. Intersectionality as a Framework for Analyzing LGBTIQ Asylum-Seekers’ Health

The existing literature suggests that an intersectionality framework is useful to analyze health inequalities affecting LGBTIQ asylum-seekers. Rooted in black feminist scholarship [38], intersectional approaches compel researchers to consider how interconnected structures of privilege and oppression (re)produce health disparities between and within groups, for instance, along the lines of migration status, ethnicity, gender, and social class [39,40]. From this perspective, the health of LGBTIQ asylum-seekers is shaped by an interaction of structural determinants on the interface of migration-related, racialized, and gender-based social dynamics.

Our study aims to provide the first insights into the health and healthcare needs of LGBTIQ asylum-seekers. In Berlin, the only major shelter for LGBTIQ asylum-seekers in Germany opened in 2016 [41]. We conducted a survey on the health status, determinants of health, access to and utilization of healthcare among the residents of Berlin’s LGBTIQ shelter. To investigate whether LGBTIQ asylum-seekers have particular healthcare needs due to intersecting determinants of health, we collected and compared similar data from asylum-seekers living in other shelters. Ultimately, our study is intended to begin filling the void on LGBTIQ asylum-seekers’ health in Germany, to support evidence-informed policies, and to instigate further research on LGBTIQ migrants’ health. 

## 2. Materials and Methods 

### 2.1. Study Design and Sampling

A cross-sectional survey was conducted among asylum-seekers from the LGBTIQ shelter and 21 other shelters in Berlin. The target population was defined as asylum-seekers who live in a shared accommodation center, are of legal age, and can complete the questionnaire in one of the nine languages provided (see Section 2.2). The LGBTIQ shelter was chosen purposefully in order to include LGBTIQ asylum-seekers in the survey; that is, the sampling of the LGBTIQ participants was non-random. The other accommodation centers were included via a weighted random sampling procedure. Using a complete list of Berlin’s accommodation centers, the facilities were divided into three categories, according to their capacity (small = <250 persons, medium = 250–500 persons, large = >500 persons). The distribution of all asylum-seekers across the different categories was calculated (19% of the population in small shelters, 59% in medium shelters, and 22% in large shelters) and corresponding numbers of accommodation centers were drawn. The management of sampled accommodation centers was contacted via email and telephone. When no contact could be established or participation in the survey was rejected, a new accommodation center was drawn. Within each accommodation center, the research team endeavored to obtain the highest possible number of respondents (cluster random sampling).

### 2.2. Instrument and Measures

We used a questionnaire that had been developed by a research team from the University Clinic Heidelberg within the framework of the RESPOND project (“Improving regional health system responses to the challenges of migration through tailored interventions for asylum-seekers and refugees”) [7]. Based on items from validated instruments, the questionnaire was designed to assess asylum-seekers’ health and healthcare. We shortened the instrument from 68 to 55 items on (i) sociodemographics, (ii) health, (iii) healthcare utilization, (iv) healthcare access, and (v) social determinants of health:

(i) Sociodemographic information comprises age, gender, legal status, length of stay in Germany, and the highest level of formal education accomplished.
(ii) Health measures include subjective health status (measured on a Likert scale from 1 = very good to 5 = very bad), a binary assessment of chronic illness *(“Do you have any longstanding illnesses or health problems?”*) taken from the European Health Interview Survey (EHIS) [42], and screening items for depression (PHQ-2) and anxiety (GAD2) [43].
(iii) Utilization of health services (general practitioner, specialist, psychotherapy/psychiatric care, hospitalization, emergency care) was assessed by means of EHIS items; namely, a binary variable (utilization/no utilization within the last 12 months) and a continuous variable (number of visits within the last 12 months).
(iv) Access to healthcare was examined by means of adapted EHIS items. Two items asked about forgone general practitioner (GP) and specialist visits in the previous year *(“During the past 12 months, was there any time when you really needed to consult a GP/specialist but did not?”*). Another item measured the prevalence of ambulatory care-sensitive (ACS) hospitalizations by asking the respondent whether she/he had been hospitalized at least once for one of 15 ACS conditions (selected from a literature-based list, [44]) in the last 12 months. Moreover, respondents were asked about out-of-pocket expenses for prescribed medicines in the past month.
(v) Measures of the social determinants of health included quality of life, social environment, and subjective social status. Quality of life was measured by means of the EUROHIS-QoL [45], a five-point, eight-item index score comprised of questions concerning overall life satisfaction *(“How satisfied are you with your life?”*), satisfaction with health *(“How satisfied are you with your health?”*), with oneself and personal relationships (“*How satisfied are you with yourself/your personal relationships?”*), and with the material situation (*“How satisfied are you with the conditions of your living place?”, “Do you have enough money to meet your needs?”*). Social environment indicators included three questions for family status taken from the EHIS (*“What is your civil status?”, “If you have a partner/spouse, is he/she currently living with you?”, “How many children do you have?”*), as well as the question *“How many people are so close to you that you can count on them if you have a serious personal problem?”*.Subjective social status was measured on a MacArthur scale (1 = bottom, 10 = top), a widely used tool with tested validity in diverse contexts [46,47,48]. Respondents were asked to rank their social status separately for the country of origin and the host country (*“Please think of your situation in Germany: In our society, there are groups which tend to be towards the top and groups which tend to be towards the bottom. At the top are the people who are best off – those who have the most money, the highest education, the most respected jobs. At the bottom are the people who are the worst off – who have the least money, least education and the least respected jobs or no job. Below is a scale that runs from the top to the bottom. Where would you put yourself on this scale? […] Now please think of your country of origin: In your country of origin, there are groups…”*).

The questionnaire and accompanying material was available in nine languages (Albanian, Arabic, English, Farsi, French, German, Russian, Serbian, and Turkish). It was handed out on paper for independent completion.

### 2.3. Setting: The LGBTIQ Shelter in Berlin

The LGBTIQ asylum-seeker accommodation center in Berlin opened in February 2016, after the Berlin Senate recognized LGBTIQ asylum-seekers as a particularly vulnerable group, and laid out specific protective measures in its integration concept [49]. It is the only accommodation center for LGBTIQ asylum-seekers in Germany, apart from one small shelter (with room for 10 persons) in Nuremberg. Funded by the State Office for Refugee Affairs, the shelter is run by the non-governmental organization “Schwulenberatung” (“Counselling for Gays”). It consists of a town house with 28 apartments, which can accommodate up to 122 persons. At the time of the survey, 80 asylum-seekers lived in the shelter. More than half of the residents identify as gay men, and 10% as lesbian women; about one-third describe themselves as “transgender, non-binary, genderfluid or queer, with the majority of them being trans*women” [41]. Two social workers and three social assistants provide social services in full-time employment. Organizations from the LGBTIQ spectrum offer further assistance, including legal counselling, psychosocial support, and health prevention. The shelter collaborates closely with a general practitioner who is sensitized to LGBTIQ health and specializes in the medical treatment of trans*persons. Through this collaboration, it is possible to offer in-house visits, as well as needs-based referrals to other healthcare providers [41].

### 2.4. Data Collection

Data collection took place between June 2018 and December 2019. The LGBTIQ shelter was visited on four days in two consecutive weeks in December 2019. A common room within the shelter was used to administer the survey. The research team set up a table, offering tea, snacks, and symbolic giveaways irrespective of study participation. It invited residents of the shelter who passed by or used the common room to participate in the survey. Study information was provided verbatim and in writing, together with the questionnaire and a stamped envelope. Filled-out questionnaires could be returned in three different ways—most (90%) were handed over in person, but participants could also submit them to a closed box that was left with the social workers, or mail them by post.

### 2.5. Sample

A total of 722 questionnaires were handed out. N = 309 valid questionnaires were returned, corresponding with approximately 8% of eligible asylum-seekers living in accommodation centers in Berlin at the time. Thirty-two participants were from the LGBTIQ shelter. The cooperation rate was 40% in the LGBTIQ shelter and 22% in the other shelters. We also included partly filled-out questionnaires in the analysis. Therefore, for some analyses, different subsample sizes are reported. 

### 2.6. Data Analysis

The reliability of the Quality of Life index measure was assessed by calculating Cronbach’s alpha; it was found internally consistent, with a reliability coefficient of 0.83. 

We examined differences in health status, healthcare access, and utilization between asylum-seekers from the LGBTIQ shelter and asylum-seekers from other shelters. To this end, we used Fisher’s Exact test for categorical variables and the Mann–Whitney U test for continuous variables (with levels of significance set at 5%). 

To test associations between residence in the LGBTIQ shelter and mental healthcare utilization, we used a generalized linear mixed model (GLMM, [50]), in which we included mental healthcare utilization as the outcome variable; mental health need and residence in the LGBTIQ shelter as fixed effects; and length of stay in Germany as a random effect. Mental health need was defined as having a score above the cut-off value for depression and/or anxiety in the questionnaire’s mental health screening items. 

Similarly, we tested associations between residence in the LGBTIQ shelter and ambulatory healthcare utilization by means of a GLMM, with ambulatory healthcare utilization as the outcome variable, chronic disease and residence in the LGBTIQ shelter as fixed effects, and subjective health status as a random effect.

A full-null model comparison was conducted for each model, with the full version of each model containing the intercept and all fixed and random effects, whereas in the null model, residence in the LGBTIQ shelter was removed as a fixed effect. To test for differences between the full and null model, we applied likelihood ratio tests [51].

All statistical analyses were performed in R 3.6.2 [52].

### 2.7. Ethical Aspects

The research team made efforts to account for the vulnerable situation of the study population. The implementation of the study was coordinated with the State Office for Refugee Affairs and with the shelters’ management and staff. Before beginning the data collection, the team introduced themselves in person to staff and residents of the shelter during an in-house plenary session, provided information and answered questions. Signs were posted to announce the study. During data collection, the researchers provided information materials in nine languages. They tried to ensure a non-threatening atmosphere and avoid any (real or presumed) pressures to participate in the study; for example, by emphasizing that participation/refusal to participate would not have any effect on asylum procedures or other personal benefits. All data were collected anonymously. Study results were discussed with residents and staff of the LGBTIQ shelter, as well as the State Office for Refugee Affairs. Ethical clearance was obtained from the Ethics Committee of the Charité University Hospital Berlin (EA4/111/18).

## 3. Results

### 3.1. Sociodemographic Characteristics and Social Determinants of Health

Respondents from the LGBTIQ shelter were younger and had higher levels of formal education as compared to asylum-seekers from other shelters. On average, they had arrived more recently in Germany than asylum-seekers from other shelters, and the majority of them still awaited a decision on their asylum request. Respondents from the LGBTIQ shelter rated their quality of life at a lower level than respondents from other shelters. 

Loneliness is common in both groups, with over one-third of respondents noting that they had no one to count on. Across both groups, many respondents regarded their subjective social status as low, both in their country of origin and in Germany (see Table 1). 

### 3.2. Health Status

Subjective health status assessments are similar for the two groups. However, chronic conditions and mental health issues are significantly more prevalent among asylum-seekers from the LGBTIQ shelter (see Table 2).

### 3.3. Healthcare Utilization and Access to Care

Healthcare utilization rates are consistently higher among asylum-seekers from the LGBTIQ shelter across all investigated types of health services. A significantly higher proportion of asylum-seekers from the LGBTIQ shelter have used ambulatory care (GP and/or specialist), mental healthcare (psychotherapy and/or psychiatric care), and emergency room services at least once within the recent year, as compared to asylum-seekers from other shelters (see Table 3). 

Respondents from the LGBTIQ shelter report significantly higher numbers of emergency room visits per year, as compared to respondents from other shelters (1 ± 1.4 as compared to 0 ± 1.1 (median ± mean absolute deviation), *p* = 0.003, 95% CI [−1; <−0.01]), as well as significantly higher numbers of hospitalizations per year (1 ± 2.0 as compared to 0 ± 1.4 (median ± mean absolute deviation), *p* = 0.024, 95% CI [−1; <−0.01]).

Over one-third of the respondents from the LGBTIQ shelter report ambulatory care-sensitive hospitalizations. In both groups, more than half of the respondents had foregone a visit to a GP or specialist at least once in the past year (see Table 3). Respondents further recount monthly out-of-pocket expenses for prescribed medications of over 83 Euro among the LGBTIQ group, and 36 Euro among asylum-seekers from other shelters. 

Respondents with a mental health need who lived in the LGBTIQ shelter were significantly more likely to use the mental healthcare system as compared to asylum-seekers from other shelters. Among chronically ill respondents, no difference in ambulatory health service utilization was found (see Table 4).

The results from our multivariate analysis indicate that residence in the LGBTIQ shelter is associated with a significantly higher probability of obtaining mental healthcare when the mental health need is controlled for (full-null model comparison GLMM χ2 = 17.215, df = 1, *p* < 0.001, for full model summary, see Table S1, supplementary material). Similarly, residence in the LGBTIQ shelter is associated with a significantly higher probability of obtaining ambulatory care, when chronic illness and subjective health is controlled for (full-null model comparison GLMM χ2 = 4.103, df = 1, *p* = 0.043, for full model summary, see Table S2, supplementary material).

## 4. Discussion

### 4.1. LGBTIQ Asylum-Seekers’ Health in Berlin—No Happy Ending, but a Silver Lining

The aim of this study was to provide preliminary evidence on the health and healthcare utilization of LGBTIQ asylum-seekers in Berlin. The Berlin authorities recognized the special needs of this group, inter alia, by establishing a separate accommodation center for them. However, scarce evidence on healthcare needs among LGBTIQ asylum-seekers sets limits on further evidence-informed policies. Following an intersectionality approach, we assumed that LGBTIQ asylum-seekers face increased health risks due to intersecting determinants of health, including migration- and gender-related marginalization. 

Indeed, our results point to a high burden of chronic and mental illness among asylum-seekers from the LGBTIQ shelter, both in comparison to asylum-seekers from other shelters, and when comparing our unadjusted averages to representative information on the German population. Among the German population, 25.3% rate their subjective health as “fair/bad/very bad”; 36.9% report a chronic illness [53]. The prevalence of depression and anxiety is 10.1% and 15.3%, respectively [54]. Even when considering that LGBTIQ persons in the German population may present with worse health outcomes, the prevalence rates for chronic and mental illness in our sample of LGBTIQ asylum-seekers (62% and 70%, respectively) still exceed the above estimates by far. Our findings thus support the hypothesis that the health of LGBTIQ asylum-seekers is under excess strain, as migration- and gender-related stressors and risks compound each other. 

At the same time, our results indicate that asylum-seekers from the LGBTIQ shelter utilize healthcare more frequently than asylum-seekers from other shelters, across all types of health services examined. As a matter of fact, their ambulatory healthcare utilization lies close to the respective rate reported for the German population (87%, [55]). Residence in the LGBTIQ shelter is associated with significantly higher odds of obtaining ambulatory and mental health services. Asylum-seekers with mental health needs who live in the LGBTIQ shelter are significantly more likely to use psychotherapy or psychiatric healthcare than asylum-seekers with mental health needs from other shelters. 

One possible explanation is that the LGBTIQ shelter offers a relatively wide range of social services. The shelter’s close collaboration with a sensitized GP may be particularly helpful in supporting access to care, as the literature indicates that primary caregivers can play an important role in facilitating asylum-seekers’ successful navigation of the healthcare system [8,56,57]. Hence, our results indicate that tailored health and support services yield some success in facilitating needs-based health service utilization. 

However, our study also shows high rates of emergency room utilization and ambulatory care-sensitive (ACS) hospitalizations among the LGBTIQ group, irrespective of their use of ambulatory and mental healthcare. By way of comparison, average emergency room utilization in OECD countries is estimated at 31% [58], and the prevalence of ACS hospitalizations at 20–27% [44]. Together with that, our results on foregone doctor’s visits and out-of-pocket payments for prescribed medicines suggest that access barriers to healthcare persist, despite the support offered in the LGBTIQ shelter. 

Hence, alternative explanations for these healthcare utilization patterns are that a) those asylum-seekers who find their way to ambulatory and mental health services are also more likely to find their way to emergency and hospital-based care; and/or b) that particularly complex health needs among LGBTIQ asylum-seekers exceed the capacities of the ambulatory healthcare provided. Alongside other factors, these unmet needs may contribute to frequent emergency room visits and hospitalizations, on top of ambulatory and mental healthcare utilization. These alternative explanations do not diminish the importance of tailored health and support services. Rather, they indicate that further research is needed to understand particular health needs and healthcare-seeking among LGBTIQ asylum-seekers, and thus enable the fine-tuning of these services. In line with the existing literature on LGBTIQ health [22,25] and asylum-seeker health [6,7,59], the areas of mental and chronic healthcare merit special attention.

In conclusion, our preliminary results indicate that the special health and social support services offered by the LGBTIQ shelter facilitate more adequate healthcare utilization, to some extent. The adjustment, strengthening, and extension of similar social and health services to all accommodation centers, akin to the “Bremen model” [8], should be considered in order to contribute to comprehensive improvements in healthcare provision for all asylum-seekers. 

Our findings further indicate that, beyond healthcare, asylum-seekers’ social conditions are not conducive of good health. The respondents in our study rate their quality of life as poor, as compared to European adults generally (3.68) and to people with depression (2.84) [45,60]. Many respondents experience loneliness, and most consider themselves at the bottom of the social ladder. Assessments are even lower for asylum-seekers from the LGBTIQ shelter, despite relatively extensive support structures. Such stressors have been shown to fundamentally impact physical and mental health [61]. The relevance of “minority stress” for the health of LGBTIQ populations has been documented [62,63], alongside the beneficial potential of social support and inclusion [31]. Hence, our study indicates that, beyond the positive measures that were taken by Berlin’s authorities, further improvements of the social determinants of asylum-seekers’ health are warranted. To enable systemic positive change, federal legal frameworks need to be adjusted toward equal social rights and inclusion of asylum-seekers.

### 4.2. LGBTIQ Asylum-Seekers’ Health as a Case-Study for Intersectionality in Migrant Health

Overall, our findings lend empirical support to intersectionality as a useful framework for migrant health research for two reasons: First, intersectionality encourages us to consider the complex dynamics between systemic marginalization and privilege, as they both drive health inequities. Second, intersectionality draws attention to inter-group heterogeneities and inequities [40]. LGBTIQ asylum-seekers are a pertinent case in point: On the one hand, interlocking structures of migration- and gender-related adversity marginalize them both within the host society and within asylum-seeking communities, thus putting their health at excess risk. On the other hand, recognition as LGBTIQ in the given policy context endows them with certain privileges, including extended health and social support services. Further research on LGBTIQ asylum-seekers’ health from an intersectionality perspective therefore has the potential to contribute to an empirically grounded theorization of how multiple, and at times, contradictory social forces become embodied in health (inequities). Moreover, the change of perspective from micro- to macro-level determinants of health and from deficits and disadvantage to (also) assets and opportunities may be an important step to move migrant health scholarship forward [16,64].

### 4.3. Limitations

Given the limited scope of our study, the findings reported here have preliminary character. Our study identified LGBTIQ asylum-seekers and refugees through a proxy, namely, their accommodation in the LGBTIQ shelter. Collinearity between LGBTIQ identity and between residence in the LGBTIQ shelter entails that effects related to openly identifying as a LGBTIQ asylum-seeker and effects related to the shelter’s extended medical and social services cannot be disentangled. In addition, we cannot rule out that the group described here as “asylum-seekers from other shelters” includes LGBTIQ individuals, who did not disclose themselves as such by describing their gender as “other”. Data collection relied on respondents’ recollection; this may induce recall bias. More detailed health measures would have allowed for more nuanced insights into the respondents’ health needs; for example, a differentiation of chronic conditions and the inclusion of stress in the mental health screening instrument (as in the DASS-21 scale). The small sample size for the LGBTIQ group limited our options for statistical analysis. 

The limitations of our study could be overcome if sexual identity and orientation were accounted for in future health research and monitoring. At the same time, this would contribute to the visibility and inclusion of LGBTIQ-specific issues in migrant health research. Further research is needed to understand the intersecting determinants of LGBTIQ asylum-seekers’ health and healthcare access, including migration-related, racialized, gender-based, and other forms of discrimination. Ideally, such research should involve community representatives as partners in the investigation of problems and in the development and implementation of solutions.

## 5. Conclusions

Our study indicates that LGBTIQ asylum-seekers face a high burden of chronic and mental illness. Living in the LGBTIQ shelter is associated with a significantly higher probability of using ambulatory and mental healthcare. Compared to asylum-seekers from other shelters, residents of the LGBTIQ shelter have a significantly higher chance of obtaining psychotherapy or psychiatric care in case of need. This may indicate that the special health and support services in Berlin’s LGBTIQ shelter help the residents, to some extent, to obtain healthcare in line with their needs. These services should be stepped up and scaled up to realize their potential and mitigate health inequities affecting asylum-seeking populations. 

However, our study also pinpoints high emergency-room utilization among the LGBTIQ group. Together with results indicating accessibility issues, such as high rates of foregone doctor visits and a high prevalence of ambulatory care-sensitive hospitalizations, this suggests that considerable unmet healthcare needs remain. The frequent utilization of in-patient and emergency care warrants further research on LGBTIQ asylum-seekers’ health. Such research should include LGBTIQ asylum-seekers’ perspectives. 

The preliminary findings reported here underlie various limitations. To generate more robust evidence on the healthcare needs of LGBTIQ asylum-seekers, the availability of data must be improved by accounting for sexual identity and orientation in future health research and monitoring.

## Figures and Tables

**Table 1:** Sociodemographic characteristics and social determinants of health.

	Asylum-Seekers from LGBTIQ Shelter (N = 32)		Asylum-Seekers from Other Shelters (N = 277)	
**Gender**				
Female	30%		34%	
Male	44%		65%	
Other/Diverse	26%		1%	
n	27		244	
**Age** (in years, mean ± SD)	29.5 ± 7		34.8 ± 12	
n	27		211	
**Highest level of education**				
no formal education	5%		34%	
mandatory schooling	27%		20%	
high school	68%		46%	
n	22		223	
**Months in Germany** (median ± meanAD)	15 ± 22		36 ± 25	
n	24		189	
**Residence status**				
Asylum-seeker	64%		35%	
Asylum granted	32%		47%	
Asylum claim rejected	4%		18%	
n	28		232	
**Quality of lifescore** (mean ± SD, 1 = low to 5 = high)	1.7 ± 0.9		2.2 ± 0.9	
n	25		263	
**Respondents with “no one to count on”**	41%		36%	
n	27		250	
**Subjective social status**	**Home country**	**Germany**	**Home country**	**Germany**
low	50%	76%	40%	56%
middle	33%	12%	26%	29%
high	17%	12%	34%	15%
n	29		255	

**Table 2:** Health status (in % (n) of respondents).

	Asylum-Seekers from LGBTIQ Shelter	Asylum-Seekers from other Shelters	Odds Ratio [95% CI]
**Good/Very good overall subjective health**	59% (17)	51% (130)	0.8 [0.31; 1.74]
n	29	255	
**Chronic illness**	63% (17) *	38% (94)	2.8 [1.15; 7.13]
n	27	249	
**Screened positive for depression and/or anxiety**	70% (19) **	34% (78)	4.5 [1.79; 12.47]
n	27	227	

* *p*-Value < 0.05, ** *p*-Value < 0.001.

**Table 3:** Healthcare Utilization and Access (in % (n) of respondents reporting at least one visit/occurrence in the previous 12 months).

	Asylum-Seekers from LGBTIQ Shelter	Asylum-Seekers from Other Shelters	Odds Ratio [95% CI]
**Ambulatory care (GP and/or specialist)**	83% (19) *	56% (130)	3.71 [1.18; 15.46]
n	23	232	
**Psychotherapy/Psychiatric care**	78% (18) **	19% (42)	15.1 [5.04; 55.05]
n	23	221	
**Emergency room**	62% (16) *	34% (80)	3.12 [1.27; 8.09]
n	26	234	
**Hospitalization**	41% (13)	24% (66)	2.14 [0.92; 4.85]
n	32	277	
**Ambulatory care-sensitive hospitalization**	36% (10)	19% (47)	2.30 [0.89; 5.68]
n	28	248	
**Foregone GP and/or specialist visit**	58% (11)	57% (118)	1.01 [0.36; 3.04]
n	19	207	

* *p*-Value < 0.05, ** *p*-Value < 0.001.

**Table 4:** Healthcare utilization among respondents with chronic and mental health needs (in % (n)).

**Respondents with a Mental Health Need**			
	**from the LGBTIQ Shelter** **(N = 15)**	**from Other Shelters** **(N = 61)**	**Odds Ratio** **[95% CI]**
**Psychotherapy/Psychiatric Care**	87% (13) **	30% (18)	14.9 [2.94; 149.5]
**Respondents with a Chronic Illness**			
	**from the LGBTIQ Shelter** **(N = 17)**	**from Other Shelters** **(N = 94)**	
**Ambulatory Care**	65% (11)	64% (60)	3.4 [0.45; 157.6]

** *p*-Value < 0.001.

## Supplementary Materials

The following are available online at https://www.mdpi.com/1660-4601/17/12/4514/s1, Table S1: Generalized Linear Mixed Model examining the association between living in the LGBTIQ shelter and the probability of ambulatory healthcare utilization, with chronic disease as fixed effect and subjective health status as random effect (n = 236), Table S2: Generalized Linear Mixed Model examining the association between residence in the LGBTIQ shelter and the probability of mental healthcare utilization, with mental health need as fixed effect and length of stay in Germany as random effect (n = 160).
