# Supplementary material for: Health and Healthcare Utilization among Asylum-Seekers from Berlin’s LGBTIQ Shelter: Preliminary Results of a Survey
Source: Int J Environ Res Public Health. 2020 Jun 23;17(12):4514. doi: 10.3390/ijerph17124514 (PMC7344488; doi:10.3390/ijerph17124514)
Supplement: Supplementary file 1 [file ijerph-17-04514-s001.pdf]

Health and Healthcare Utilization among Asylum-Seekers from Berlin's LGBTIQ shelter: Preliminary Results of a Survey - Supplementary material

Nora Gottlieb [1*], Conny Püschmann [2], Fabian Stenziger [2], Julia Koelber [2], Laurette Rasch [2], Martha Koppelow [2] and Razan Al Munjid [2]

[1] Department of Health Care Management, Technical University Berlin, 10623 Berlin, Germany
[2] Berlin School of Public Health, 10117 Berlin, Germany; conny.pueschmann@charite.de (C.P.); fabian.stenzinger@charite.de (F.S.); julia.koelber@charite.de (J.K.); laurette.rasch@charite.de (L.R.); martha.koppelow@charite.de (M.K.); razan.almunjid@charite.de (R.A.-M.)
* Correspondence: nora.gottlieb@tu-berlin.de; Tel.: +49 (0)30 314 29222

Table S1: Generalized Linear Mixed Model examining the association between living in the LGBTIQ shelter and the probability of ambulatory healthcare utilization, with chronic disease as fixed effect and subjective health status as random effect (n = 236)

Term	Estimate	Std.Error	z-value	95% CI	χ^2	df	p-Value
Intercept	–2.086	0.634	–3.219	–3.462;−0.915			
LGBTIQ shelter	1.144	0.607	1.885	0.035; 2.470	4.103	1	0.043
Chronic disease	1.075	0.361	2.979	0.363; 1.781	8.714	1	0.003
Random effect: Subjective health	0.146	0.383					

Table S2: Generalized Linear Mixed Model examining the association between residence in the LGBTIQ shelter and the probability of mental healthcare utilization, with mental health need as fixed effect and length of stay in Germany as random effect (n = 160*)*

Term	Estimate	Std.Error	z-value	95% CI	χ^2	df	p-Value
Intercept	–0.689	0.417	–1.652	–1.588; 0.142			
LGBTIQ shelter	2.692	0.732	3.678	1.387; 4.360	17.215	1	<0.001
No mental health need	–1.221	0.495	–2.466	–2.281; -0.288;	6.613	1	0.010
Random effect: Length of stay	1.308	1.144					
